# Population genomic variation reveals roles of history, adaptation and ploidy in switchgrass

**DOI:** 10.1111/mec.12845

**Published:** 2014-07-21

**Authors:** Paul P Grabowski, Geoffrey P Morris, Michael D Casler, Justin O Borevitz

**Affiliations:** * U.S. Dairy Forage Research Center, USDA-ARS1925 Linden Drive, Madison, WI 53706, USA; † Department of Ecology and Evolution, University of Chicago1101 East 57th Street, Chicago, IL 60637, USA; ‡ Department of Agronomy, Kansas State University3004 Throckmorton Plant Sciences Center, Manhattan, KS 66506, USA; § Research School of Biology, Australian National UniversityLinnaeus Building #134, Canberra, ACT 0200, Australia

**Keywords:** ecotypes, genotyping-by-sequencing, perennial grass, polyploidy

## Abstract

Geographic patterns of genetic variation are shaped by multiple evolutionary processes, including genetic drift, migration and natural selection. Switchgrass (*Panicum virgatum* L.) has strong genetic and adaptive differentiation despite life history characteristics that promote high levels of gene flow and can homogenize intraspecific differences, such as wind-pollination and self-incompatibility. To better understand how historical and contemporary factors shape variation in switchgrass, we use genotyping-by-sequencing to characterize switchgrass from across its range at 98 042 SNPs. Population structuring reflects biogeographic and ploidy differences within and between switchgrass ecotypes and indicates that biogeographic history, ploidy incompatibilities and differential adaptation each have important roles in shaping ecotypic differentiation in switchgrass. At one extreme, we determine that two *Panicum* taxa are not separate species but are actually conspecific, ecologically divergent types of switchgrass adapted to the extreme conditions of coastal sand dune habitats. Conversely, we identify natural hybrids among lowland and upland ecotypes and visualize their genome-wide patterns of admixture. Furthermore, we determine that genetic differentiation between primarily tetraploid and octoploid lineages is not caused solely by ploidy differences. Rather, genetic diversity in primarily octoploid lineages is consistent with a history of admixture. This suggests that polyploidy in switchgrass is promoted by admixture of diverged lineages, which may be important for maintaining genetic differentiation between switchgrass ecotypes where they are sympatric. These results provide new insights into the mechanisms shaping variation in widespread species and provide a foundation for dissecting the genetic basis of adaptation in switchgrass.

## Introduction

A major goal of evolutionary biology is to understand how genetic variation is generated, shaped and maintained within a species. Gene flow and recombination act to homogenize a species, while a myriad of processes, including mutation, range shifts and natural selection, counteract those homogenizing forces to generate genetic differences (Hewitt 1996; Nosil *et al*. 2009; Novembre & Di Rienzo 2009). Many widespread, outcrossing plant species have strong population structure (Lowry *et al*. 2008; Eckert *et al*. 2010; Zalapa *et al*. 2011) despite the potential for high levels of gene flow to homogenize genomic differences throughout these species (Keller *et al*. 2010; Prunier *et al*. 2011), making these species appealing systems for understanding how different mechanisms can oppose gene flow to generate spatial and genome-wide patterns of variation within a species.

Diversity in widespread plants is affected by multiple factors, each of which leaves a signature on patterns of intraspecific variation. For instance, many widespread species underwent major range expansions and contractions during the Pleistocene glacial cycles, and contemporary population structure often reflects this biogeographic history, such as genetic divisions or geographic patterns that originated from these historical range shifts (Hewitt 1996; Soltis *et al*. 2006). Adaptive differences can also greatly influence patterns of variation, particularly in species with different ecotypes, where differential adaptation can reduce gene flow via immigrant and hybrid inviability and thus promote genetic differentiation between ecotypes (Porter 1966; Lowry *et al*. 2008). Additionally, in many plants, genomic events such as polyploidy and chromosomal rearrangements are associated with intra- and interspecific reproductive isolation and differentiation (Rieseberg *et al*. 1995; Soltis *et al*. 2007; Lowry & Willis 2010).

Polyploidy is of particular interest because of the major impact it can have on gene flow in a species. Multiple ploidy levels exist within many species, and gene flow between ploidy levels is restricted by the low fitness of the progeny produced by crosses between ploidy levels (Martinez-Reyna & Vogel 2002; Henry *et al*. 2005). This reproductive isolation occurs immediately upon polyploidization; thus, polyploidy can generate differentiation independent of geographic allopatry (Coyne & Orr 2004). In addition, physiological and genetic effects of polyploidy may directly result in adaptive differentiation within a species (Otto & Whitton 2000). However, the role of polyploidy in shaping intraspecific diversity is poorly understood because ploidy differences often correlate with adaptive and geographic patterns, and distinguishing the individual effects of each factor on patterns of diversity remains a major challenge (Levin 1983; Ramsey & Schemske 2002). There may also be some aspects of polyploidy that can only be better understood by incorporating information about the other factors that shape diversity. For example, competition between cytotypes and fitness costs from interploidy matings favour the exclusion of new cytotypes (Levin 1975; Petit *et al*. 1999); therefore, other factors may have key roles in the establishment and maintenance of multiple cytotypes, which is seen in many species. Thus, by characterizing the association of species-wide population structure with ploidy, geography, phenotype and habitat, we can assess the effects of the multiple factors that affect gene flow and examine how they combine to generate patterns of variation.

In the perennial North American grass switchgrass (*Panicum virgatum* L.), ploidy is one of the many factors that shape variation across its extensive native range. Switchgrass is a foundational species for habitat restoration, emerging bioenergy feedstock and forage crop (Sanderson *et al*. 1996), and the resources developed for these purposes, including extensive germplasm collections, make switchgrass an attractive system for examining how diversity is shaped throughout a widespread species. Switchgrass is divided into two clades: the lowland and upland ecotypes, which are genetically distinct despite substantial sympatry in their distributions (Zhang *et al*. 2011b). The geographic distribution of genetic differences between and within the ecotypes suggests that switchgrass was periodically partitioned into separate refugia during glacial maxima, with some genetic differences resulting from those periods of allopatry persisting until today (McMillan 1959; Zhang *et al*. 2011b). The lowland and upland ecotypes are adapted to different edaphic conditions and have low viability in the habitat of the other ecotype (Porter 1966), and throughout the species, there is strong local adaptation to growing season (McMillan 1964). In addition, switchgrass has multiple ploidy levels (Costich *et al*. 2010), and a ploidy-related incompatibility system restricts gene flow between ploidy levels (Martinez-Reyna & Vogel 2002). The lowland ecotype is primarily tetraploid while the upland ecotype has both tetraploid and octoploid lineages (Costich *et al*. 2010); thus, ploidy differences may be important in shaping differences within and between switchgrass ecotypes. Furthermore, switchgrass hybridizes with its sister species, *Panicum amarum* and *P. amarulum*, in portions of the range (Palmer 1975; Barkworth *et al*. 2007). However, the roles and relative importance of these different factors in shaping the overall diversity in switchgrass remain poorly understood. For instance, ploidy may have a major impact on diversification and the patterns of gene flow in switchgrass, but key details about ploidy are not known, such as the timing of polyploidization in different lineages or the frequency of natural ploidy shifts. Thus, it is difficult to decipher the effect of ploidy on generating and maintaining diversity in the species.

Recent studies examining genomic diversity in switchgrass have begun to better resolve patterns of population structure (Morris *et al*. 2011; Lu *et al*. 2013). However, these studies have focused primarily on the upland ecotype, so patterns of genomic diversity across the species and within the lowland ecotype have been largely unexplored. Therefore, we use genotyping-by-sequencing (GBS; Elshire *et al*. 2011) to genotype more than 100 switchgrass samples from across the US range, as well as three related *Panicum* taxa at 98 042 SNPs. With these data, we can better understand how geography, adaptive differences, and ploidy shape diversity throughout this ecologically and economically important grass.

## Materials and methods

### Plant materials

To survey species-wide patterns of diversity in switchgrass, we genotyped 123 *Panicum virgatum* samples from 41 populations (Table1; Table S1, Supporting information). The switchgrass plants used in this study represent collections from remnant habitats and from cultivated varieties which are seed increases of source-identified collections that have undergone few generations of amplification and are considered good representations of the native genetic variation from original collection locations (Zalapa *et al*. 2011). These samples included two dihaploids, which have only one copy of each chromosome from each subgenome (Young *et al*. 2010) and should have no heterozygosity. We also included one sample each of *P. amarum*, *P. amarulum* and *P. anceps*. The *P. amarum* and *P. amarulum* lineages are alternatively referred to as either separate species or subspecies of the same taxon (*P. amarum*; Barkworth *et al*. 2007; Triplett *et al*. 2012), and their taxonomic definition is further complicated by reports of hybridization between *P. amarulum* and switchgrass (Palmer 1975). We included the *P. amarum* and *P. amarulum* samples, as well as a sample from a more distantly related species, *P. anceps* (Aliscioni *et al*. 2003), to assess their relationships with switchgrass and as outgroups for the within-switchgrass comparisons.

**Table 1:** Population information

Population name	*n*	State	Latitude	Longitude	Inferred gene pools*	Population type†	Seed source‡
Rocky Run 1	2	WI	43.47	−89.43	Upland Northern Great Plains	Wild	SW112
Hwy 59	4	WI	42.9	−87.55	Upland Northern Great Plains	Wild	SW127
Bald Bluff	3	WI	42.85	−88.63	Upland Northern Great Plains	Wild	SW128
Staten Island	5	NY	40.5859	−74.1482	Lowland Atlantic Coastal Plain;Upland Midwest;Upland Eastern Savanna	Wild	SW781
Blackwell	7	OK	35.963	−97.07	Upland Northern Great Plains	SIC	PI 421520;ECS
Cave-in-Rock	6	IL	37.47	−88.1658	Upland Eastern Savanna;Mixed Upland	SIC	PI 469228;ECS
Rt 72/563 NJ	4	NJ	39.817	−74.533	Upland Northern Great Plains;Upland Eastern Savanna;Mixed Upland	Wild	ECS-1
Howard	1	IN	40.4508	−86.1281	Upland Northern Great Plains	Wild	SW33
Waterford	2	WI	42.78	−88.3	NA	Wild	SW123
NRCS 9064224	1	IN	40.481	−86.22	Mixed Upland	Wild	NRCS-PMC
Columbiana	1	OH	40.616	−80.695	Upland Eastern Savanna	Wild	SW64
Wadena	1	MN	46.4433	−95.1349	Upland Northern Great Plains	Wild	SW60
Chiwaukee 1	1	WI	42.55	−87.8	Upland Midwest	Wild	SW124
NRCS 9084291	2	MI	42.983	−86.059	Upland Midwest	Wild	NRCS-PMC
Dacotah	3	ND	46.3845	−100.9398	Upland Eastern Savanna	SIC	NRCS-PMC
Pathfinder	2	KS	39.82	−98.48	Upland Northern Great Plains	SIC	USDA-ARS
Shelter	4	WV	39.396	−81.199	Upland Eastern Savanna;Mixed Upland	SIC	NRCS-PMC
Sunburst	3	SD	42.872	−97.3957	Upland Northern Great Plains	Bred	SDCIA;ECS
Toledo, OH	1	OH	41.583	−83.667	Upland Northern Great Plains	Wild	ECS-2
Allegheny River, PA	1	PA	40.95	−79.617	Upland Eastern Savanna	Wild	ECS-10
Hoffman	1	NC	35.0306	−79.5456	Lowland/Upland Hybrid	Wild	PI 315723
Sprewell Bluff	3	GA	32.899	−84.436	Lowland/Upland Hybrid	Wild	UGA-SPB
Kanlow	7	OK	35.3288	−96.2408	Lowland Southern Great Plains	SIC	PI 421521
AW-314/MS-155	1	AR	35.4266	−91.836	Lowland Southern Great Plains	Wild	PI 421999
PMT-785	4	TX	29.443	−96.94	Lowland Western Gulf Coast	Wild	PI 422003
T 2086	3	NC	34.2358	−77.9412	Lowland Atlantic Coastal Plain	Wild	PI 476290
Oscar Scherer S.P.	2	FL	27.1859	−82.4565	Lowland Atlantic Coastal Plain	Wild	UGA-OSP
Pasco County	1	FL	28.33	−82.42	Upland Midwest	Wild	UGA-PCF
Wabasso	2	FL	27.747	−80.435	Lowland Atlantic Coastal Plain	Wild	PI 422000
Chippewa	3	MN	45.52	−95.307	Upland Northern Great Plains	Wild	SW48
Jackson	3	MI	42.2537	−84.3101	Upland Midwest	Wild	SW43
Ipswich Prairie 2	4	WI	42.57	−90.4	Upland Midwest	Wild	SW115
Albany, NY	4	NY	42.717	−73.833	Upland Northern Great Plains	Wild	ECS-12
Pangburn	5	AR	35.4266	−91.836	Lowland Southern Great Plains	Wild	PI 414065
BN-12323-69	5	KS	38.81	−98.27	Upland Midwest	Wild	PI 414070
Panicum amarum	1	NA	NA	NA	Lowland	Wild	NA
Panicum amarulum	1	NA	NA	NA	Lowland Atlantic Coastal Plain	Wild	NA
Panicum anceps	1	NA	NA	NA	NA	Wild	NA
Alamo	1	TX	28.3305	−98.1163	Lowland Southern Great Plains	SIC	ECS
Indiana Dunes S.P.	16	IN	41.6582	−87.0577	Upland Midwest	Wild	PPG-IDSP
Forestburg	1	SD	44.022	−98.105	Upland Northern Great Plains	SIC	ECS
Shawnee	1	IL	37.47	−88.1658	Upland Eastern Savanna	Bred	ECS
Southlow	1	MI	NA	NA	Mixed Upland	Ecopool	NRCS-PSMC

* Based on samples above read-count cut-off.

† Bred, a product of one or more cycles of selection and breeding; SIC, source-identified cultivar derived from a random seed increase without selection and breeding; Wild, seed harvested from remnant population.

‡ USDA-ARS, switchgrass breeding programme (Lincoln, NE); SDCIA, South Dakota Crop Improvement Association (Brookings, SD); NRCS-PMC, NRCS Plant Materials Centers (Bismarck, ND; Rose Lake, MI; Big Flats, NY; Cape May, NJ; Americus, GA; Coffeeville, MS); PI-xxxxxx, NRCS-GRIN; Germplasm Resources Information Network, USDA-ARS (Beltsville, MD); ECS-xx, Ernst Conservation Seeds (Meadville, PA); SWxxx, seeds collected directly from prairie remnant site and processed in Madison, WI; UGA-xxx, seeds collected directly from prairie remnant site and processed in Athens, GA; PPG-IDSP, seeds collected directly from remnant habitat and processed in Chicago, IL.

### Ploidy and phenotypic data

Switchgrass is allopolyploid with a base chromosome count of 9, and the two most common ploidy levels are tetraploid (2*n* = 4X = 36) and octoploid (2*n* = 8X = 72; Costich *et al*. 2010). Tetraploid switchgrass shows disomic inheritance and can be genotyped like a diploid, with three genotype classes of AA, AB, BB (Okada *et al*. 2010). Octoploid switchgrass has sets of four homologous chromosomes and therefore has five potential genotype classes of AAAA, AAAB, AABB, ABBB and BBBB. Ploidy data were previously obtained using flow cytometry for a majority of the samples (Zhang *et al*. 2011a; Table S1, Supporting information). For others, ploidy information was inferred from previously characterized cultivars with well-known and stable ploidy levels (Costich *et al*. 2010; Zalapa *et al*. 2011; Lu *et al*. 2013). For the IDSP population, ploidy was inferred from flow cytometry of a daughter plant of one sample (D. Lowry, personal communication). We do not have flow cytometry data for the three other *Panicum* samples. *Panicum amarum* and *P. amarulum* are generally hexaploid and tetraploid, respectively, but there is evidence of multiple ploidy levels in these lineages (Triplett *et al*. 2012). The phenotypes of the samples were characterized as ‘lowland ecotype’, ‘upland ecotype’ or ‘intermediate’ based on traits that are characteristic of each ecotype, including plant architecture and leaf colour (Zhang *et al*. 2011a).

### Genotyping-by-sequencing

Seven pooled genotyping-by-sequencing lanes, each composed of 36 multiplexed samples, were sequenced on an Illumina GA-IIx sequencer (Illumina, San Diego, CA, USA) using 100 bp paired-end reads. Technical replicates of each sample were sequenced in separate libraries. GBS library preparation was adapted from Elshire *et al*. (2011), using PstI as the restriction enzyme. Additional information about DNA extraction, library preparation and sequencing adapters is in the supporting material. One subtle but important improvement in library construction was to amplify and normalize individual libraries before pooling and sequencing. This reduced the range in coverage per sample by nearly an order of magnitude.

Custom R-scripts (R Development Core Team 2011) were used to process and assign sequencing reads to the appropriate sample based on the sample-specific DNA barcode at the beginning of each first-end sequencing read. Any reads containing sequence from the sequencing adapters or PCR primers were omitted. Candidate SNPs were identified according to Morris *et al*. (2011). Any loci with SNPs at three or more consecutive positions likely contain indels and were removed. We also excluded any SNPs more than 50 bp from a restriction site to remove potential artefacts due to lower quality at the ends of sequencing reads. To address challenges associated with measuring relatedness in a species with multiple ploidy levels, we generated pseudo-haploid genotypes by randomly sampling one sequencing read for each sample at each locus, and calling the genotype based on the allele in that sequencing read, as previously described (Morris *et al*. 2011). For computational reasons, a randomized subset of the GBS loci was used for the pseudo-haploid genotypes, and the pseudo-haploid genotypes are used for all analysis using genetic distances.

Tetraploid switchgrass has disomic inheritance (Okada *et al*. 2010), so we also generated diploid genotypes to estimate levels of heterozygosity and visualize genome-wide patterns of admixture in hybrid samples, with the understanding that heterozygosity estimates will be increased in octoploids because they contain twice as many alleles of each locus as do the tetraploids. We used the following cut-offs for assigning diploid genotypes: heterozygous A:B genotypes require at least one read each of allele A and allele B. Homozygous A:A genotypes require six or more reads of allele A without any reads of allele B, otherwise they are assigned a partial, A:*NA* genotype.

### Population structure

Population structure was investigated with principal coordinate analysis (PCoA) using the *dist.dna* (‘ape’ package, Paradis *et al*. 2004) and *cmdscale* functions in R (R Development Core Team 2011) using pseudo-haploid genotypes. Pseudo-haploid genotypes were also used to generate neighbour-joining trees using the *bionj* function (‘ape’ R package, Paradis *et al*. 2004). We also used structure (Pritchard *et al*. 2000) to assess population structure and admixture using pseudo-haploid genotypes. For runs using all loci, we used a 500 k-cycle burn-in followed by 2 million MCMC cycles. We also generated five random subsets of 1000 loci and used a 100k-cycle burn-in followed by 500 k MCMC cycles. We followed Evanno *et al*. (2005) to determine the number of demes best supported by the results. Isolation by distance was evaluated by calculating the full and partial correlations of genetic distance and log-transformed geographic distance in R (R Development Core Team 2011), including ploidy, ecotype and/or regional gene pool as covariates.

### Genome-wide patterns of admixture

To visualize the genome-wide patterns of admixture in identified hybrid samples, we mapped the GBS loci from this analysis to the *Setaria italica* reference genome (Bennetzen *et al*. 2012) using 80% homology across 50% of the GBS sequence (clc_assemble_ref_long, CLC Assembly Cell, CLC bio, Cambridge, MA, USA), and retaining only loci with unique hits to the *S. italica* reference. Grasses have high levels of conserved synteny (Devos 2005), and *S. italica* is closely related to switchgrass (Bennetzen *et al*. 2012), so the genomic location of the loci mapped to *S. italica* should closely resemble their relative location in the switchgrass genome. Because *S. italica* is diploid while switchgrass is polyploid, homeologous switchgrass GBS loci will map to the same location in the *S. italica* genome, thus partially masking the true patterns of admixture. Therefore, this method provides a preview of the possibilities when using the assembled switchgrass genome that is in progress. Alleles were considered private in an ecotype if the SNP is genotyped in at least 10 individuals in the focal ecotype and is either completely absent or genotyped in at least 10 individuals in the other ecotype without seeing the allele.

### Estimating genome-wide nucleotide diversity

The pseudo-haploid genotypes are equivalent to the genotype of a single copy of the genome within each sample, so genetic distance represents the amount of differences between two copies of the genome. We used the average pairwise genetic distance within a population as an estimate of the genome-wide level of nucleotide diversity in a population and therefore as a way to compare relative levels of diversity. This raw diversity quantification is another advantage of GBS data over predefined SNP data. We calculated average within-population genetic distance for all populations with more than one sample using *dist.dna* (‘ape’ package, Paradis *et al*. 2004) in R (R Development Core Team 2011). Populations that contained samples from different gene pools based on population structure results were divided into subgroups based on gene pool. We did not include samples that show a strong signal of admixture between regional gene pools within each ecotype based on PCoA results.

### Simulated octoploids

To test whether differences in heterozygosity between tetraploid and octoploid groups are due strictly to their different number of chromosomes, we generated simulated octoploid genotypes by combining diploid genotypes of two samples from the primarily tetraploid gene pools. We used the following rules: the 8X genotype is heterozygous if both alleles are found in either sample. The 8X genotype is homozygous if both samples are homozygous or partial (e.g. A:*NA*) for the same allele. The 8X genotype has a partial genotype if one sample is partial and the other has no genotype. We simulated within-gene pool octoploids using all pairwise combinations of samples within each tetraploid gene pools and admixed octoploids using all pairwise combinations of samples from different tetraploid gene pools.

## Results

### Sequencing and genotyping results

We generated genotyping-by-sequencing libraries for 126 samples, producing 141.8 million paired-end sequence reads on the Illumina GA-IIx platform. Of these reads, 132.8 million (93.6%) passed preliminary filtering criteria. The mean per-sample read count was 1.05 million reads, but varied from 53 243 to 2 802 834 reads per sample (Table S1, Supporting information). We removed 14 samples with less than 310 000 sequencing reads from further analysis, as these had an excess of SNPs with missing data (Fig. S1a, Supporting information), and because above that threshold, diversity measures are minimally affected by sequencing coverage (Fig. S1c, Supporting information). We identified 98 042 SNPs on 27 666 short contigs (called GBS loci in the rest of the text). Of these, 71 020 (72.4%) SNPs are genotyped in at least 80 samples, 19 182 (19.6%) are genotyped in all the switchgrass samples and 3549 (3.6%) are also genotyped in the *Panicum amarum*, *P. amarulum* and *P. anceps* samples. There are 6896 (7.0%) SNPs where the minor allele is found in a single individual, with the number of singleton SNPs per sample ranging from 2 to 252 in the switchgrass samples, and 763, 58 and 399 in the *P. amarum*, *P. amarulum* and *P. anceps* samples, respectively. Our ability to detect singletons is reduced as we require significant variation in allele counts across samples due to a chi-squared filter we apply to our genotyping results (as in Myles *et al*. 2010; Morris *et al*. 2011). For computational reasons, 19 907 SNPs (20.3%) were randomly selected to use for the pseudo-haploid genotypes in all the individuals.

Using the dihaploid samples, we identify 9.2% (9016) of the putative SNPs representing coassembled homeologs rather than alleles at single genomic loci. The dihaploid samples are both part of the same gene pool, lowland Southern Great Plains and therefore cannot identify homeologous SNPs that are variable only in other gene pools. Therefore, we retain the homeologous SNPs in the analysis because removing them would disproportionately remove variation from the lowland Southern Great Plains gene pool and bias diversity measures. However, PCoA results are qualitatively the same whether the detected homeologous SNPs are included or removed.

### Population structure

**Figure 1:**
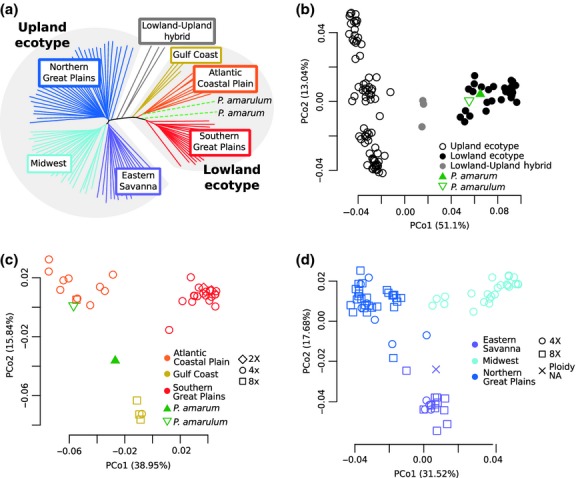
Population structure in switchgrass inferred from neighbour-joining tree (a) and PCoA (b, c, d). Colour corresponds to inferred ecotype (b) or regional gene pool (a, c, d), and shape corresponds to ploidy (c, d). The *Panicum amarum* and *P. amarulum* samples are labelled individually (a–c), and their ploidy in unknown. (a) Neighbour-joining tree of all switchgrass samples, *P. amarum* and *P. amarulum*. (b) PCoA with samples from (a). Note that *P. amarum* and *P. amarulum* cluster with lowland ecotype samples. (c) PCoA with lowland ecotype samples, *P. amarum* and *P. amarulum* shows three lowland regional gene pools. (d) PCoA with upland ecotype samples shows three upland regional gene pools.

**Figure 2:**
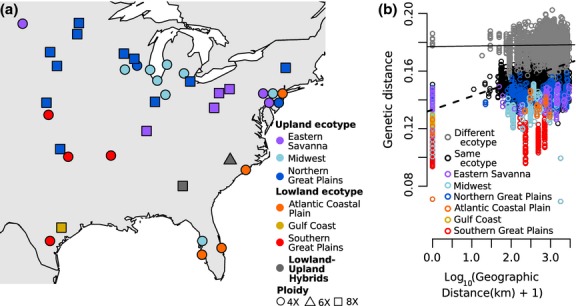
Geographic patterns of genetic diversity. (a) Map of populations. Colour corresponds to inferred regional gene pool, and shape corresponds to predominant ploidy. (b) Patterns of isolation by distance shown by all pairwise comparisons of genetic and geographic distance. Grey = between ecotypes. Black = within ecotype. Colours = within each regional gene pool. Lines representing the linear regression of all between-ecotype comparisons (solid line, slope = 3.5 × 10^−4^) and all within-ecotype comparisons (dashed line, slope = 9.4 × 10^−3^) indicate greater IBD within ecotypes than between ecotypes.

To characterize species-wide patterns of population structure and genome-wide nucleotide diversity, we used pseudo-haploid genotypes to evaluate the relationships of switchgrass from 41 populations across the United States. PCoA and structure resolve two main groups corresponding to the upland and lowland switchgrass ecotypes (Fig.1b; Figs S2a, S3c, S4, Supporting information), as expected (Morris *et al*. 2011; Zalapa *et al*. 2011). We further detect additional population stratification within both major switchgrass ecotypes. Within the lowland ecotype, three major gene pools are resolved (Fig.1a,c; Fig. S2b, Supporting information) corresponding to three US geographic areas: the Southern Great Plains, the Western Gulf Coast and the Atlantic Coastal Plain (Fig.2a). While the Western Gulf Coast gene pool is represented by a single population, the log-likelihood values from structure are consistent with three demes, and McMillan (1964) also identified a morphologically distinct group in the same region. Within the upland ecotype, three main gene pools are identified, as well, (Fig.1a,d; Fig. S2c, Supporting information) corresponding to the Northern Great Plains, the Midwest, and the Eastern Savanna of the United States (Fig.2a). These gene pools correspond with the three upland clades identified by Lu *et al*. (2013). Several upland samples cluster between gene pools (Table S1, Supporting information; Fig.1d), and these sample are removed when calculating average genetic distances within populations and between gene pools.

The lowland and upland ecotypes remain genetically distinct across their large and partially overlapping ranges (Fig.2a), as the geographic distance to the most-distant sample of the same ecotype ranges from 1500 to 3000 km, while the distance to the closest sample of the other ecotype ranges from 0 to 500 km. Similarly, there is only a minimal relationship between genetic and geographic distances of samples from different ecotypes (Fig.2b). A pattern of isolation by distance (IBD, *r* = 0.356, Fig.2b, Table3) in the data set is driven largely by IBD within the gene pools, as the signal of IBD decreases dramatically when accounting for regional gene pools (*r* = 0.169, Table3).

Each switchgrass gene pool is primarily one ploidy level, either tetraploid (4X) or octoploid (8X). However, ploidy is not fixed within the gene pools, as the three primarily 8X gene pools contain at least one 4X sample (Fig.1c,d). In addition, two 8X samples that did not pass the read-count cut-off are part of populations in the primarily 4X gene pools and cluster with those gene pools when included in the analysis (Fig. S3d,e, Supporting information). Octoploid samples are more similar to tetraploids from the same gene pool than to octoploids from other gene pools (Fig.1), indicating that ploidy has changed multiple times in switchgrass. Lu *et al*. (2013) concluded that the upland ecotype was ancestrally octoploid and a haploidization event generated the tetraploid upland Midwest gene pool. However, the presence of tetraploids in all three upland gene pools (Fig.1) suggests that, instead, the upland ecotype was ancestrally tetraploid and that octoploidy arose independently in the upland Eastern Savanna and Northern Great Plains gene pools. Furthermore, as no gene pool is exclusively octoploid, then polyploidy could not have initiated the differentiation between gene pools, nor are any gene pools completely reproductively isolated due to ploidy differences.

### *Panicum amarum* and *P. amarulum* are ecotypes of *P. virgatum*

We included *P. amarum* and *P. amarulum* samples as outgroups for our switchgrass analysis and to evaluate their relationship to switchgrass, as they are generally considered a sister lineage to *P. virgatum* (Barkworth *et al*. 2007). However, the *P. amarum* and *P. amarulum* samples in this study cluster within the lowland ecotype (Fig.1b; Fig. S4, Supporting information), as also seen using gene trees (Triplett *et al*. 2012). Furthermore, *P. amarum* and *P. amarulum* are genetically more similar to the lowland ecotype samples than to the upland ecotype samples (Table2). These results are in contrast to the *P. anceps* sample that clusters separately from all the other samples (Figs S3b and S4, Supporting information). These results indicate that *P. amarum* and *P. amarulum* are, in fact, switchgrass ecotypes adapted to the harsh conditions of coastal habitats (Barkworth *et al*. 2007). Interestingly, while *P. amarum* and *P. amarulum* are often considered part of the same taxon, the *P. amarulum* in this study is more similar to the lowland Atlantic Coastal Plain gene pool than it is to the *P. amarum* sample (Fig.1c, Table2), supporting the hypothesis that *P. amarum* and *P. amarulum* are not part of a single lineage that is distinct from the lowland and upland switchgrass lineages (Triplett *et al*. 2012). While these patterns need to be confirmed with more samples, they indicate that *P. amarulum* is a morphologically diverged type of the switchgrass lowland Atlantic Coastal Plain gene pool, or potentially the result of hybridization between the Atlantic Coastal Plain gene pool and the *P. amarum* ecotype (Palmer 1975; Barkworth *et al*. 2007).

**Table 2:** Average pairwise genetic distance of gene pools and *Panicum* samples

	Upland Midwest (24)	Upland Northern Great Plains (32)	Upland Eastern Savanna (15)	Lowland Atlantic Coastal Plain (9)	Lowland Southern Great Plains (18)	Lowland Western Gulf Coast (4)	Hybrid-SPB (2)	Hybrid-HOF (1)	*P. amarulum* (1)	*P. amarum* (1)
Upland Midwest										
Upland Northern Great Plains	0.1518									
Upland Eastern Savanna	0.1482	0.1529								
Lowland Atlantic Coastal Plain	0.1825	0.1794	0.1788							
Lowland Southern Great Plains	0.1821	0.1773	0.1791	0.15						
Lowland Western Gulf Coast	0.1742	0.1676	0.1699	0.1558	0.1425					
Hybrid-SPB	0.1605	0.1592	0.158	0.1572	0.1596	0.1545				
Hybrid-HOF	0.1621	0.1559	0.1615	0.1623	0.1581	0.1591	0.1603			
*P. amarulum*	0.1748	0.1707	0.1715	0.1293	0.1479	0.1472	0.1489	0.1559		
*P. amarum*	0.1856	0.182	0.1824	0.153	0.1503	0.1485	0.1629	0.1713	0.1434	
*P. anceps* (1)	0.3498	0.345	0.3551	0.3595	0.3314	0.346	0.3492	0.3448	0.356	0.3421

Parentheses indicate the number of samples included in each group. The lowland–upland hybrids are separated into population of origin. Note lowland Western Gulf Coast is more similar to upland ecotype gene pools than are either other lowland ecotype gene pools.

### Hybridization and gene flow

**Figure 3:**
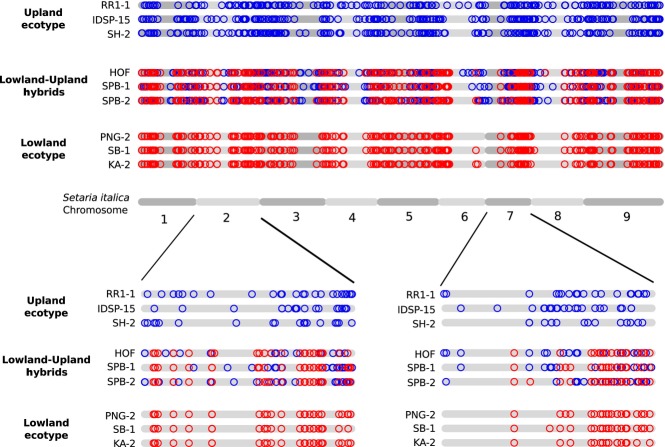
Genome-wide distribution of ecotype-specific alleles in lowland–upland hybrids. Alleles private to either ecotype are mapped to the reference genome of *Setaria italica*. Patterns are shown in the three identified lowland–upland hybrid samples as well as three upland ecotype and three lowland ecotype samples. Red = lowland ecotype allele. Blue = upland ecotype allele. To normalize for the overall higher number of upland private alleles in the data set (due to the higher number of upland vs. lowland samples), a random subset of 25% of upland alleles is plotted. Genomic regions of predominantly upland or lowland ancestry can be identified. Magnifications of chromosomes 2 and 7 show chromosome-wide patterns at higher resolution.

There is a large overlap in the ranges of the lowland and upland switchgrass ecotypes, but reports of natural hybrids between the ecotypes are rare (Zhang *et al*. 2011a), despite the close proximity of many upland and lowland switchgrass populations (Fig.2). However, we identify three samples that group between the lowland and upland ecotype clusters in the PCoA (Fig.1b), show strong patterns of lowland–upland admixture (Fig. S2a, Supporting information) and are equally related to the upland and lowland ecotypes (Table2), indicating that they are the result of recent hybridization between the ecotypes. The hybrid nature of these samples is further supported by their genome-wide distribution of ecotype-specific alleles (Fig.3) and previous results using EST-SSR markers (Zhang *et al*. 2011a). The hybrid populations are both in the Atlantic Coastal Plain (Fig.2a), and further sampling is needed to examine whether natural hybridization between the ecotypes is common in that region.

In addition to the hybrids, we also detect gene flow from the upland ecotype into the lowland Western Gulf Coast gene pool, as that gene pool has a signal of admixture with the upland ecotype (Fig. S2a, Supporting information) and is more similar to the upland ecotype than is either other lowland gene pool (Table2). These findings are supported by EST-SSR results that see lowland–upland ecotype admixture in the same population (Zhang *et al*. 2011a).

### Diversity measures indicate admixture promotes polyploidy

**Figure 4:**
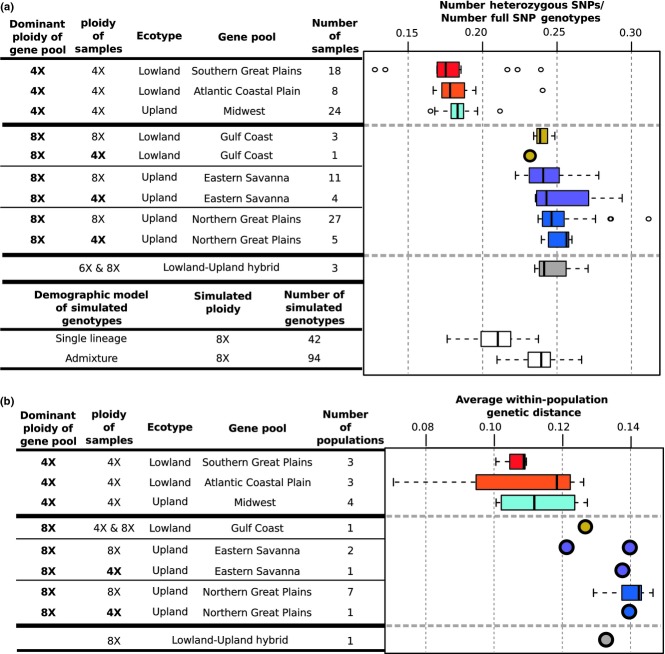
Genetic diversity of switchgrass gene pools. Samples (a) and populations (b) from gene pools with both 4X and 8X samples are divided into subgroups by ploidy. (a) Number of heterozygous SNPs divided by the number of SNPs with full diploid genotype calls as an estimate of heterozygosity for each sample. Simulated octoploid genotypes were generated by combining genotypes from tetraploids either from the same gene pool (‘Single lineage’) or from the different gene pools (‘Admixture’). Heterozygosity levels in predominantly 8X gene pools are more similar to the admixed rather than the single-lineage simulated octoploids. (b) Average within-population genetic distance as an estimate of nucleotide diversity. Note that heterozygosity (a) and nucleotide diversity (b) are the same for both 4X and 8X samples from primarily 8X gene pools. Also, primarily 8X gene pools have similar diversity levels as natural (lowland–upland hybrids) and simulated admixed samples.

The gene pools have different levels of estimated heterozygosity and nucleotide diversity corresponding to their primary ploidy levels, with the primarily 8X gene pools having higher levels of both measures than the 4X gene pools (Fig.4). However, the tetraploid members of the primarily 8X gene pools also have increased levels of both heterozygosity and nucleotide diversity (Fig.4), indicating that the increased diversity measures in the primarily 8X gene pools are not exclusively due to higher ploidy. To estimate how diversity measures would be affected by a shift from tetraploid to octoploid, we simulated octoploid genotypes by combining data from tetraploid samples in the same gene pool. The simulated single-lineage octoploids have lower heterozygosity than the true octoploid gene pools (Fig.4), further indicating that ploidy alone does not explain the increased diversity in the primarily 8X gene pools.

Interestingly, the upland–lowland hybrid samples have similar levels of nucleotide diversity and heterozygosity as the primarily 8X gene pools (Fig.4), and one primarily 8X gene pool, lowland Western Gulf Coast, shows evidence of admixture between the lowland and upland ecotypes (Table2; Fig. S2a, Supporting information). This suggests that increased diversity in the primarily 8X gene pools may be due to admixture. Therefore, to estimate how a combination of admixture and octoploidy would affect diversity, we simulated admixed octoploid genotypes by combining tetraploid samples from different gene pools. The simulated admixed octoploids have heterozygosity similar to the levels seen in the primarily 8X gene pools (Fig.4a), supporting a hypothesis that the primarily 8X gene pools have a history of admixture. Results using gene trees are also consistent with octoploid switchgrass being derived from the combination of two distinct tetraploid lineages (Triplett *et al*. 2012).

## Discussion

Geospatial and genome-wide patterns of nucleotide diversity provide a window into the historical and contemporary processes that shape variation in a species (Novembre & Di Rienzo 2009). Thus, to characterize how different processes have contributed to differentiation in switchgrass, we genotyped 123 switchgrass individuals from 41 populations across the US range at 98 042 SNPs. The major genetic division detected within switchgrass is between the lowland and upland ecotypes, consistent with previous results (Fig.1b, Morris *et al*. 2011; Zalapa *et al*. 2011; Lu *et al*. 2013), and we also identify three regional gene pools within both ecotypes (Fig.1a,c,d). Patterns of ploidy within and between gene pools (Fig.1c,d) indicate that the upland and lowland ecotypes were ancestrally tetraploid (4X) and that octoploidy (8X) has independently arisen multiple times. In addition, we detect signatures of historical and contemporary hybridization between the upland and lowland ecotypes (Fig.1b; Fig. S2a, Supporting information; Table2) and determine that *Panicum amarum* and *P. amarulum* are actually switchgrass ecotypes and not separate taxa (Fig.1a,b,c; Table2). Furthermore, increased diversity in the primarily octoploid gene pools is not solely the result of polyploidy, but rather is consistent with a history of admixture in these gene pools.

### Biogeographic history, adaptation and ploidy shape switchgrass variation

We detect three regional gene pools within both the upland and the lowland ecotypes (Fig.1). Each gene pool has a dominant ploidy, either tetraploid (4X) or octoploid (8X); however, samples of the alternate ploidy are seen in several gene pools, including all three primarily 8X gene pools (Fig.1; Fig. S3, Supporting information). The three upland gene pools correspond to the three main upland clades identified by Lu *et al*. (2013). Lu *et al*. (2013) propose that the upland ecotype was ancestrally octoploid and that the upland tetraploid gene pool underwent haploidization to become tetraploid and therefore reproductively isolated from the other upland lineages. However, the presence of tetraploids in the upland Eastern Savanna and Northern Great Plains gene pools instead indicates that the upland ecotype was ancestrally tetraploid and octoploids arose independently in the two primarily 8X gene pools.

Because tetraploids are present in all the observed switchgrass gene pools, it is unlikely that polyploidy itself generated the genetic differences seen in switchgrass. Instead, geography was likely the main driver of differentiation in switchgrass. The geographic patterns of population structure are consistent with the hypothesis that switchgrass was restricted to multiple refugia during glacial maxima (McMillan 1959; Zalapa *et al*. 2011; Lu *et al*. 2013). For example, the geographic distribution of the lowland ecotype gene pools (Fig.2a) corresponds with predicted distinct glacial refugia in Florida and along the Gulf Coast (McMillan 1959; Zalapa *et al*. 2011) and corresponds with phylogeographic patterns seen in other species (Soltis *et al*. 2006). Similarly, the genetic differentiation and geographic distribution of upland gene pools suggest that the upland ecotype was restricted to multiple distinct glacial refugia, as well (Fig.2a). Ploidy differences between gene pools likely arose subsequent to genetic divergence due to vicariance.

Despite this historical vicariance within both ecotypes, populations of the same ecotype that were in different refugia are more closely related than are populations of different ecotypes just metres away (Fig.2). Similarly, when accounting for the spatial structure of the gene pools within each ecotype, there is little isolation by distance (Table3), indicating that one or more mechanisms are acting to restrict gene flow between the ecotypes, resulting in the observed range-wide differentiation between ecotypes.

**Table 3:** Correlations of genetic distance and geographic distance

*r*-Value	Lower (2.5%) confidence limit	Higher (97.5%) confidence limit	Ecotype	Regional gene pool	Ploidy
0.356	0.326	0.386			
0.371	0.340	0.397	X		
0.169	0.145	0.192		X	
0.342	0.311	0.374			X
0.250	0.230	0.275	X	X	
0.360	0.329	0.392	X		X
0.172	0.150	0.197		X	X
0.251	0.228	0.275	X	X	X

Correlation and partial correlation coefficients of genetic distance and log-transformed geographic distance. Xs indicate the covariate(s) used when calculating partial correlations. Confidence limits based on 500 bootstrap replicates.

In many species, differential adaptation restricts gene flow between ecotypes via immigrant and hybrid inviability (Lowry *et al*. 2008). The lowland and upland ecotypes are adapted to different edaphic conditions and have lower fitness in the habitat of the other ecotype (Porter 1966), suggesting that differential adaptation may restrict gene flow in switchgrass, as well. Most populations show little signature of gene flow from the other ecotype (Fig. S2a, Supporting information), even when in close proximity to populations of the other ecotype. However, we do detect gene flow from the upland ecotype into the lowland Western Gulf Coast gene pool (Table2) as well as contemporary hybridization between the lowland and upland ecotypes (Fig.3; Fig. S2, Supporting information), showing that adaptive differences are not sufficient to restrict all gene flow between the ecotypes and the low levels of inter-ecotype gene flow in most samples are unlikely due solely to differential adaptation.

While intraploidy gene flow is at least possible between any gene pool of either ecotype (Fig.1; Fig. S3, Supporting information), in many areas inhabited by both ecotypes, particularly the Great Plains, the primary ploidy level of the lowland and upland ecotypes differs (Figs1 and 2, Zhang *et al*. 2011b). As noted by Lowry *et al*. (2014), these ploidy differences may help to restrict contemporary gene flow between the ecotypes in these areas. In fact, ploidy differences acting to prevent maladaptive hybridization and maintain differentiation between ecotypes may be a mechanism common to many species (Martin & Husband 2013).

### Admixture promotes polyploidy in switchgrass

Within both the lowland and upland switchgrass ecotypes, the frequency of the major ploidy levels differs substantially between genetically distinct regional gene pools (Fig.1c,d). In many plants, hybridization and admixture are associated with subsequent higher rates of polyploidy (Ramsey & Schemske 1998; Chapman & Burke 2007; Paun *et al*. 2009; Parisod *et al*. 2010). Interestingly, we see a similar pattern in switchgrass. Increased ploidy is seen in lineages with signals of admixture between the lowland and upland ecotypes (Fig.1; Fig. S2a, Supporting information; Table2). In the upland gene pools with prevalent octoploidy, we do not detect genetic signals of admixture between ecotypes (Fig. S2a, Supporting information), but there is indication that these lineages may have a history of admixture, as well. First, tetraploid samples within the upland primarily 8X gene pools have the same levels of diversity as the octoploid samples (Fig.4), so increased diversity in these lineages is not due strictly to higher ploidy. Second, the Eastern Savanna and Northern Great Plains gene pools have similar levels of heterozygosity and nucleotide diversity as the lowland–upland hybrids and admixed lowland Western Gulf Coast gene pool (Fig.4). Third, the levels of heterozygosity seen in the Eastern Savanna and Northern Great Plains gene pools are only replicated in synthetic admixed octoploids and not in synthetic octoploids made using samples from the same gene pool (Fig.4a). Furthermore, gene tree analysis indicates that upland octoploids are derived from the combination of two separate upland lineages (Triplett *et al*. 2012). Combined, these results strongly suggest that the upland primarily 8X gene pools have a history of admixture, most likely of diverged upland populations. The overall association of octoploidy with admixture suggests that polyploidization (i.e. increases in ploidy from baseline tetraploid) in switchgrass is promoted by the admixture of genetically diverged lineages, both between and within ecotypes.

Several studies show a positive link between the genetic divergence between species and the formation of polyploids upon hybridization (Chapman & Burke 2007; Paun *et al*. 2009). However, the universality of this pattern has been doubted because these studies omit polyploids that contain genome copies from only a single progenitor species, generally termed autopolyploids (Buggs *et al*. 2009; Soltis *et al*. 2010). Predating this debate, Stebbins proposed that even within species, polyploidy is often due to the interaction of historically isolated and diverged lineages (Stebbins 1984), forming what can be considered ‘intraspecific allopolyploids’ because they contain genome copies from diverged lineages but the same species. In fact, in several species, polyploidy is seen in suture zones where lineages that were previously isolated in different glacial refugia have come into secondary contact and interbred (Parisod *et al*. 2010). Our data indicate that switchgrass lineages in which octoploidy is common have a history of admixture (Fig.4). These results further support Stebbins’ secondary contact hypothesis for intraspecific polyploids and suggest that hybridization between diverged lineages increases the likelihood of polyploidy even within species.

### Interdependence of ploidy, adaptation and biogeography

In many plant species, ploidy differences are associated with adaptive differences (Parisod *et al*. 2010; Manzaneda *et al*. 2011; Martin & Husband 2013, table2 for list of examples). Polyploidy has immediate phenotypic effects (Otto 2007) and could directly result in adaptive differences, potentially because of increased genetic diversity or because genetic redundancy allows for neo- and subfunctionalization of genes for adaptive traits (Lynch *et al*. 2001). Alternatively, adaptive differences may develop unrelated to ploidy, but ploidy-related reproductive isolation could prevent the flow of maladapted alleles between differentially adapted cytotypes. Our results indicate that polyploidy was not directly associated with the adaptive differentiation of the major lowland and upland switchgrass ecotypes, although it is possible that polyploidy generates adaptive differences within the ecotypes. However, in many areas where the lowland and upland ecotypes are sympatric, the ecotypes have different ploidy (Zhang *et al*. 2011b; Lowry *et al*. 2014). Similarly, ploidy difference exists between the primarily 4X lowland Atlantic Coastal Plain switchgrass gene pool (including *P. amarulum*) and the *P. amarum* coastal ecotype, which is often hexaploid (Barkworth *et al*. 2007). Thus, in regions of sympatry, ploidy often seems to have a role in restricting gene flow between differentially adapted ecotypes.

An intriguing possibility is that the interaction of sympatric ecotypes is important for promoting the establishment and maintenance of alternate ploidy levels. The establishment of polyploids is repressed by minority cytotype exclusion and competition between ploidy levels (Levin 1975; Petit *et al*. 1999). In switchgrass, octoploidy itself has negative fitness consequences because octoploids produce high numbers of aneuploid offspring (Costich *et al*. 2010), and no adaptive advantage associated with octoploidy has been demonstrated. The negative effect from polyploidization could, however, be offset by the fitness advantage from preventing maladaptive hybridization, thereby increasing the prevalence of polyploids in these areas.

Furthermore, our results indicate that admixture promotes polyploidy in switchgrass (Fig.4) and that previously isolated switchgrass lineages have come into secondary contact possibly as a result of postglacial range expansion (Fig.2a). Thus, the biogeographic history of switchgrass may have been crucial for generating the admixed upland lineages prone to becoming octoploid and therefore able to coexist and avoid maladaptive hybridization with sympatric tetraploid lowland populations.

### Germplasm for dissecting the genetic basis of phenotypes

Adaptive differences have been studied in switchgrass for more than 50 years (McMillan 1959; Porter 1966), and recent advances in genomic methods have opened up new avenues for dissecting the genetic basis of these classic cases of ecotypic adaptation (Li *et al*. 2010; Parchman *et al*. 2012). Switchgrass is also an important foundational species for grassland restoration efforts and an emerging bioenergy feedstock, and understanding the genetic basis of adaptive traits is a key for accelerating improvement efforts, including developing genetic markers for marker-assisted selection (Varshney *et al*. 2005). Genome-wide association studies, which can use natural populations to identify common genetic variation associated with traits, are a powerful approach for dissecting the genetic basis of phenotypes, particularly for species like switchgrass where phenological differences and long generation times complicate the development of mapping populations. And with the release of a reference switchgrass genome on the horizon, genome-wide association studies will be an important part of improvement efforts in the near future.

Our study provides a foundation for future genome-wide association studies in switchgrass, such as the regional gene pools (Figs1 and 2) which need to be accounted for when designing species-wide association panels (Myles *et al*. 2009; Brachi *et al*. 2011) and are candidates for regional mapping approaches (Brachi *et al*. 2013). In addition, *P. amarum* and *P. amarulum*, which we identify as a switchgrass ecotypes, are underused resources for studying adaptation to marginal conditions in switchgrass, as they are adapted to the sand dunes along marine coasts. However, many adaptive traits are characteristic differences between the lowland, upland and coastal switchgrass ecotypes (Porter 1966; Palmer 1975), and the correlation of these traits with ecotypic genetic differentiation will confound most association studies (Myles *et al*. 2009). As such, natural hybrid populations where the intermating of the ecotypes breaks up the associations of phenotypes with population structure, such as the Hoffman and Sprewell Bluff populations identified here (Table1, Fig.3), are a valuable resource for dissecting the genetic basis of important ecotype-specific traits.

## Data accessibility

Raw sequencing reads are available at NCBI SRA under BioProject Accession PRJNA252891 (BioProject ID: 252891). Diploid genotypes, pseudo-haploid genotypes and additional data used in the analysis are available from the DRYAD repository, doi:10.5061/dryad.k77nh. Table1 contains collection locations of the samples.

## Supporting Information

Table S1 Samples, ploidy, phenotype, and sequencing output.

Table S2 Sequencing adapter and DNA barcode information.

Fig S1 Genotyping statistics compared to sequencing read count. **Fig. S2** structure results for switchgrass samples using subset of 1000 SNPs. **Fig S3** Additional PCoA plots. **Fig S4** Rooted neighbor-joining tree including all samples above the read-count cutoff.

Appendix S1 Sequencing library preparation methods.

## Abbreviations

P.P.G.: designed research, performed library preparation, analysed genetic data and wrote the paper.
G.P.M.: designed research, processed sequencing data to generate genotypes and edited the paper.
M.D.C.: provided genetic material and information about samples and edited the paper.
J.O.B.: designed research and edited the paper.
