# Supplementary material for: Population genomic variation reveals roles of history, adaptation and ploidy in switchgrass
Source: Mol Ecol. 2014 Jul 21;23(16):4059–73. doi: 10.1111/mec.12845 (PMC4142443; doi:10.1111/mec.12845)
Supplement: Fig S1 — Genotyping statistics compared to sequencing read count. Fig. S2 structure results for switchgrass samples using subset of 1000 SNPs. Fig S3 Additional PCoA plots. Fig S4 Rooted neighbor-joining tree including all samples above the read-count cutoff. [file mec0023-4059-SD3.doc]

# Supplemental Figures

## Figure S1


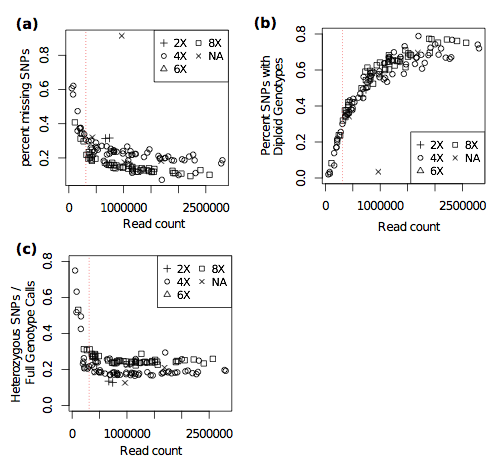


**Figure S1**: Genotyping statistics compared to sequencing read count. Samples below the 310,000 read cutoff (red, dashed line) were omitted from the rest of the analysis. **(a)** Percentage of missing SNPs **(b)** Percentage of SNPs with full, diploid genotypes **(c)** Ratio of heterozygous SNPs to SNPs with full, diploid genotypes, as an estimate of heterozygosity.

## Figure S2


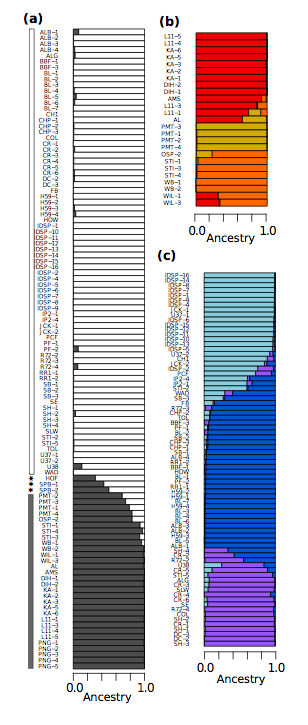


**Figure S2**: STRUCTURE results for switchgrass samples using subset of 1000 SNPs. Colors correspond to ecotype or regional gene pools. **(a)** All switchgrass samples, k = 2. Asterix indicate lowland-upland samples identified with PCoA. Gray and white boxes indicate lowland and upland ecotype samples, respectively, used in (b) and (c). **(b)** Lowland ecotype samples, k = 3. Red = Southern Great Plains; yellow = Western Gulf Coast; orange = Atlantic Coastal Plain. **(c)** Upland ecotype samples, k = 3. Purple = Eastern United States Savanna; light blue = Midwest, blue = Northern Great Plains.

## Figure S3


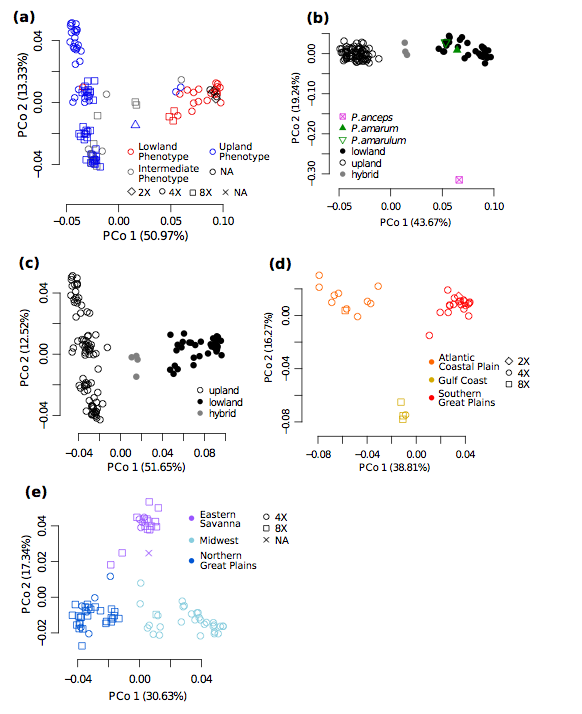


**Figure S3**: Additional PCoA plots. **(a)** PCoA of genotype of all switchgrass samples, labeled by observed phenotype (color) and ploidy (shape). Phenotypic designation does not always correspond with clustering based on genotype. **(b)** All samples above the read-count cutoff, including *P. anceps*, which clusters by itself. Labels correspond to *Panicum* taxa and, within switchgrass, ecotype. **(c)** All switchgrass samples, including samples below the read-count cutoff, labeled by ecotype. **(d)** Lowland ecotype samples, including samples below the read-count cutoff. **(e)** Upland ecotype samples, including samples below the read-count cutoff.

##
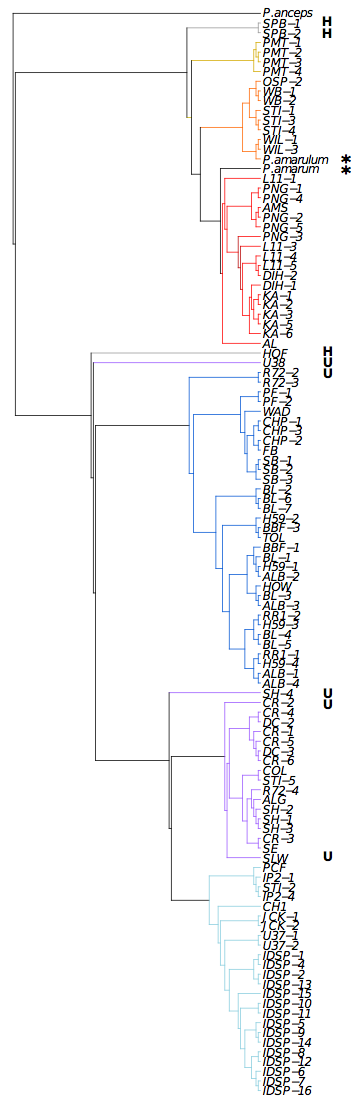
Figure S4

**Figure S4**: Rooted neighbor-joining tree including all samples above the read-count cutoff. H = Lowland-upland hybrid samples based on PCoA. U = Upland ecotype, mixed gene pools based on PCoA. * = *P. amarum* *and P. amarulum.* Line colors correspond to gene pools inferred from PCoA. In lowland ecotype clade, red = Southern Great Plains; yellow = Western Gulf Coast; orange = Atlantic Coastal Plain. In upland ecotype clade, purple = Eastern United States Savanna; light blue = Midwest; blue = Northern Great Plains. Gray = Hybrids of lowland and upland ecotypes. Note that *P. amarum* and *P. amarulum* are both in the lowland clade, but are on different branches of the tree.
