# Supplementary material for: Population genomic variation reveals roles of history, adaptation and ploidy in switchgrass
Source: Mol Ecol. 2014 Jul 21;23(16):4059–73. doi: 10.1111/mec.12845 (PMC4142443; doi:10.1111/mec.12845)
Supplement: Appendix S1 — Sequencing library preparation methods. [file mec0023-4059-SD4.doc]

## Supplemental Methods

**DNA extraction, sequencing library preparation, and next-generation sequencing**

Genomic DNA was extracted from leaf tissue using a Qiagen DNeasy kit (Qiagen, Valencia CA, USA) or a CTAB-based method adapted from Chen and Ronald (1999). DNA quality was assessed on a NanoDrop 1000 (Thermo Fisher Scientific, Waltham, MA, USA). Seven pooled genotyping-by-sequencing (GBS) lanes, each composed of 36 multiplexed samples, were sequenced.  Samples were replicated across lanes to even out the sequencing read counts and to accommodate for any failed samples during library preparation. GBS library preparation was adapted from Elshire et al. (2011). Genomic DNA for each sample was combined with 0.6 pmol each of the barcode and common sequencing adapters (Table S2). The adapter sequences are the same as Elshire et al. (2011) except for the replacement of the ApeKI sticky-end with the PstI sticky-end sequence and the alteration of some barcode sequences to ablate the PstI site after ligation. 100ng-1000ng genomic DNA was digested with 4 units of PstI-HF (NEB, Ipswitch, MA, USA) for two hours, then incubated with 640 units of T4 DNA Ligase (NEB, Ipswitch, MA, USA) at 16 degrees for 60 minutes to ligate the digested genomic DNA to the sequencing adapters.  Each sample was purified separately using the MinElute 96 UF PCR Purification Kit (Qiagen, Valencia, CA, USA) and eluted with 25ul 10mM Tris-Cl, pH 8.5. The products of the digestion and ligation were amplified in a 50ul reaction volume using 10ul of the purified ligation product as template, 1x Taq Master Mix (NEB, Ipswitch, MA, USA) and 10 pmol of each PCR primer (AATGATACGGCGACCACCGAGATCTACACTCTTTCCCTACACGACGCTCTTCCGATC*T, CAAGCAGAAGACGGCATACGAGATCGGTCTCGGCATTCCTGCTGAACCGCTCTTCCGATC*T, * = phosphorothioation).  PCR cycles were as follows: 68 degrees for 5 minutes, 95 degrees for 1 minute, 18 amplification cycles (95 degrees for 30 seconds, 65 degrees for 30 seconds, 68 degrees for 30 seconds), and 68 degrees for 5 minutes. We find that amplifying the ligation products separately, then pooling the samples, generates more consistent read-counts per sample compared to pooling samples before the PCR amplification.  For each library, 10ul of PCR-product from each sample was pooled and purified using the QIAquick PCR purification kit (Qiagen, Valencia, CA, USA) and eluted with 50 uL 10mM Tris-Cl, pH 8.5. Library quality was checked on a NanoDrop 1000 and by running 5ul of the final library on an agarose gel.

## References

Chen DH, Ronald PC (1999) A rapid DNA minipreparation method suitable for AFLP and other PCR applications. *Plant Molecular Biology Reporter*, **17**, 53–57.

Elshire RJ, Glaubitz JC, Sun Q, Poland JA, Kawamoto K, Buckler ES, Mitchell SE (2011) A robust, simple genotyping-by-sequencing (GBS) approach for high diversity species. *PLoS ONE*, **6**, e19379.
